# Hospitals and the Spirit Tax: Mr. F. A. Hocking's Proposals

**Published:** 1915-07-10

**Authors:** F. A. Hocking

**Affiliations:** Pharmaceutist. London Hospital.


### Hospitals and the Spirit Tax: Mr. F. A. Hocking's Proposals.

*To the Editor of* The Hospital.

Sir,—With respect to the recent Parliamentary discussion on the vital question of duty-free spirit for use in hospitals, I am enclosing copy of a letter I have addressed to the *Pharmaceutical Journal* upon the subject, which I think might be of interest to readers of The Hospital if space can be found for it.—Yours faithfully,

F. A. Hocking, Pharmaceutist.

London Hospital.

The following is the letter referred to (with our cross-heading), under date July 3:—

Sir,—The large amount of space devoted to "Hospitals and the Spirit Duties" render this week's *Pharmaceutical Journal* peculiarly interesting to a hospital pharmacist.

As a pharmacist who has spent twenty-five years in the service of three voluntary hospitals, and who is associated with two other hospitals in an advisory capacity, I would remark that much printers' ink and Parliamentary time would be saved if writers and speakers were to make themselves more conversant with the organisation and routine of hospital pharmacy. "Observer" conjures up visions of smuggling by out-patients. On the average an out-patient of this hospital takes away from this hospital a total of 42 minims of duty-paid spirit during his or her course of treatment. What would be the possibilities of smuggling if these 42 minims were duty free?

I enclose a card of printed instructions issued to each dispenser, specifying the exact amount of any preparation to be supplied to each patient per week. For liniments the quantity is 1 oz. if applied by a brush, 3 oz. if applied by friction. This disposes of any necessity on the part of a dispenser to ask the patient how much is required, and incidentally destroys Mr. Jones' "pint of liniment" theory.

If the public acted upon Mr. Jones' dictionary definition of potable, in respect of "all medicines," the coroners of this country would be the busiest men in England. It is a very common thing for medical men to ask a hospital pharmacist to explain why a chemist charges so very much more for drugs than a hospital pays for them. If spirit is to cost a hospital less than it costs the chemist, it would be but one more instance to explain. In view of this and many other points which might be raised, Mr. Jones' remarks on the training of medical students has little or no force.

Mr. Jones' criticisms appear to be: (1) The difficulty of defining a hospital; (2) the possibility of unlimited use of duty-free spirit; (3) the question of safeguards; (4) the question of rebate; (5) the incentive to intemperance.

How to Get Over the Spirit Duty.

(1) The difficulty of finding a definition is not insuperable.

The question of duty-free spirit has been before the voluntary hospitals for many years, and as they are the institutions most concerned, I suggest one way of getting over this difficulty would be to regard as hospitals only those institutions which are eligible to receive grants from the King Edward's Hospital Fund for London or the Metropolitan Hospital Sunday Fund, and such provincial institutions as would be, if situated in the Metropolitan area, eligible for grants. Such definition would exclude

